# Measles Outbreaks in the Republic of Congo: Epidemiology of Laboratory-Confirmed Cases Between 2019 and 2022

**DOI:** 10.1155/2024/8501027

**Published:** 2024-10-28

**Authors:** Yanne Vanessa Thiécesse Mavoungou, Fabien Roch Niama, Léa Gwladys Gangoué, Felix Koukouikila-Koussounda, Marianne Bouanga Bayonne, Cynthia Nkoua Badzi, Leblanc Albert Gandza Gampouo, Pathou Christelle Kiminou, Paule Biyama-Kimia, Princesse Mahoukou, Nadia Claricelle Bongolo Loukabou, Jean Medard Kankou, Pembe Issamou Mayengue, Gabriel Ahombo

**Affiliations:** ^1^Laboratoire National de Santé Publique, 120 Avenue Du Général Charles DE GAULE, BP: 120, Brazzaville, Congo; ^2^Direction de l'Epidémiologie et de la Lutte Contre la Maladie, Brazzaville, Congo; ^3^Faculté des Sciences et Techniques, Université Marien NGOUABI, BP: 69, Brazzaville, Congo

**Keywords:** epidemiology, measles, outbreak, Republic of the Congo

## Abstract

In Africa, measles epidemics are frequently reported, despite numerous preventive measures, such as vaccination, which targets children under 5 years of age. Unfortunately, the Republic of the Congo is not an exception to this major health concern. Indeed, many cases are reported annually. Here, we provide an overview of the epidemiological characteristics of laboratory-confirmed measles cases from January 2019 to October 2022 as well as the risk factors associated with the occurrence of measles outbreak. Samples from suspected measles cases were collected across the country and sent to the National Laboratory of Public Health for confirmation. Specific IgM was tested using the enzyme-linked immunosorbent assay (ELISA). Data were analyzed using descriptive and analytic statistics (*p* < 0.05 was statistically significant). A total of 1330 samples were collected and analyzed. Over those 4 years, 537 samples were confirmed to be positive (40.3%) but with important disparities between years. A relatively low frequency of cases was reported in 2020. Overall, a progressive and significant evolution of positive cases was observed between 2019 and 2022, increasing from 16.8% in 2019 to 65.9% in 2022 (*p* < 0.0001). We report a low vaccination rate among children (44.8%) and a significantly high positivity rate in this group (46.6%) (*p* < 0.0008). No difference was reported according to the completeness of the vaccination scheme (*p*=0.094). Females were slightly more exposed to this infection than males (*p*=0.04; adjusted odds ratio [aOR]: 1.25 [1.01–1.6]), with an increased risk of exposure in rural areas (*p*=0.0001; aOR: 0.41 [0.32–0.53]). The department of Pointe-Noire had the highest positivity rate, while three other departments were considered high-risk areas: Likouala (*p* = 0.0001; aOR: 3.18 [1.80–5.61]), Pool (*p*=0.0001; aOR: 2.90 [1.70–4.95]), and Brazzaville (*p*=0.0005; aOR: 0.52 [0.36–0.75]). This study calls for strengthening the epidemiological surveillance system and vaccination strategy in the country. It remains important to research factors that induce a high positive rate among vaccinated children by biological verification of the immunization.

## 1. Background

Measles is a highly infectious viral disease with a reproductive rate varying from 12 to 18 in populations at risk [1]. It is transmitted through air or by direct contact with the secretions of an infected person [2]. Multiple clinical signs have been reported and the most severe forms of the disease result in high fever (39°C–40°C), pneumonia, diarrhea, dehydration, vomiting, and encephalitis. This infection can lead to death, especially in children aged between 0 and 5 years [2–4].

Globally, a resurgence of measles has been observed in recent years in many countries, including those where the disease seems to have been eradicated [5]. Indeed, a significant peak in the number of confirmed cases was noted as early as 2019, a year in which 869, 770 cases were reported worldwide, which is the highest figure since 1996, according to the World Health Organization (WHO) [6]. Since then, this trend has been confirmed between the beginning of 2021 and 2022, in which an increase in cases of almost 79% was reported. In the same period, between April 2021 and April 2022, there were 21 outbreaks in Africa and the Eastern Mediterranean region [7]. This upsurge is occurring in the context of increased availability of preventive measures such as vaccination, but paradoxically, we are also witnessing a decline in the rate of vaccination coverage in different countries since 2021. To eliminate measles, the WHO recommends that countries achieve a minimum of 95% vaccination coverage, with equitable administration of different doses of the vaccine, in the hope of achieving herd immunity and stopping the circulation of the virus [8, 9]. In 2019, the average vaccine coverage for many sub-Saharan African countries did not exceed 80% [10, 11], which allows for the endemic circulation of the virus to be maintained in many countries, and measles sometimes explodes in outbreaks [12–14]. This would explain, among other things, the difficulty in eliminating this disease, given the failure of vaccination coverage, which is often suboptimal in many developing countries [3]. Since the beginning of 2022, many African countries such as Somalia, Nigeria, Mali, Ethiopia, the Democratic Republic of the Congo (DRC), and the Ivory Coast have reported measles epidemics [15]. However, one of the deadliest in the last decade occurred between 2020 and 2021 in the DRC, with more than 6000 deaths in children [16].

In the Republic of the Congo, measles has been circulating for a long time in an endemic epidemic manner. Certain health districts of the departments have become epicenters, and many cases of measles are frequently reported there. Currently, a measles epidemic is occurring in our country with the department of Pointe-Noire as the epicenter. Unfortunately, few data are currently available on the dynamics of circulation and the epidemiology of this disease in the Republic of the Congo. Since 2012, the country has subscribed to the strategic plan for measles elimination in the African region. Through its Expanded Program on Immunization, the country has implemented a multiyear plan, in which one of the strategic goals is fighting against measles, mumps, and rubella. This program introduced the MMR (measles/mumps/rubella) combined vaccine in 2015 [17]. This vaccine is administered in two doses to children aged 09 and 15 months. However, despite the efforts made, numerous difficulties, such as the geographical accessibility of certain areas, have been cited as factors that can favor the emergence of measles cases [17]. These factors limit the possibility of having comprehensive data on the measles situation in the country. In 2021, for example, a vaccination coverage rate of 80% for the first dose and less than 50% for the second dose were reported for the country [10]. These rates remain far below the 95% target recommended by the WHO. The aim of this study is to provide an overview of the epidemiological characteristics of laboratory-confirmed measles cases from January 2019 to October 2022 as well as the risk factors associated with the occurrence of measles in the Republic of the Congo.

## 2. Methods

### 2.1. Setting and Study Design

With an area of 3,42,000 km^2^, the Republic of the Congo comprises 12 departments. It is bordered to the north by the Central African Republic and Cameroon, to the south by Angola toward the Cabinda enclave and part of the DRC, to the east by the DRC, and to the west by Gabon and the Atlantic Ocean [17].

**Figure 1:**
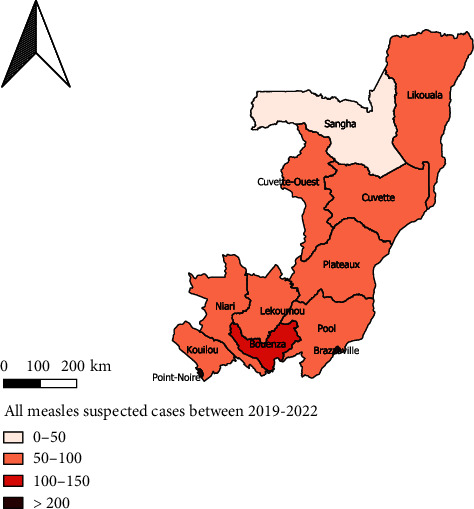
Map' settings of the study location and highlighting hotspots for all measles cases between 2019 and 2022 around all 12 departments. This map was designed with QGIS 3.16. The shapefile source was downloaded online.

All departments have been implicated in the study. In each department, a focal point has been designated. He/she is located in the department prefecture and is affiliated with the immunization program. The focal point is responsible for taking the sample and packaging it according to national standards and transferring it via a collection system setup with the support of the WHO country office to the National Public Health Laboratory for analysis [17]. In case of positive results, the sample is sent to the Pasteur Institute of Dakar in Senegal for confirmation.  Figure 1 shows not only the scope of our study but also the nuances between departments and the different hotspots from which measles cases are most routinely reported.

### 2.2. Type of Study

This is a descriptive and analytic study of all laboratory-confirmed cases after measles suspicion by the case notification system in different departments across the country from January 2019 until October 2022.

### 2.3. Case Definition

A suspected case of measles was defined as anyone with a generalized maculopapular rash and fever and any of the following symptoms: cough, coryza (runny nose), or conjunctivitis (red eyes). A measles outbreak is declared when three cases had been confirmed positive in a health zone over a health period. A laboratory-confirmed case is a suspected case whose laboratory results indicate infection by detection of Type M immunoglobulins (IgM) or isolated measles virus.

### 2.4. Sampling and Transport

Samples were collected from January 2019 to October 2022. As measles is a notifiable disease, all persons meeting the case definition were systematically included in the study. Only samples with degraded quality were not included. All samples were collected during the serological detection window. Blood samples were collected from the elbow bend in preidentified 5 mL EDTA-free tubes or in 5-mL EDTA tubes. Then, samples were first centrifuged at 4000 rpm for 5 min to obtain serum or plasma, and aliquots of samples were made in preidentified cryotubes. Then, those aliquots were transferred between 24 and 72 h into isothermal coolers and maintained at 2°C–4°C during transport.

### 2.5. Laboratory Analysis

These analyses were performed at the National Public Health Laboratory for the detection of IgM using the enzyme-linked immunosorbent assay (ELISA) technique made by the anti-measles virus ELISA (IgM kit, EUROIMMUN Lübeck, Germany). The algorithm for assay validation was as follows: the optical density (OD) of the calibrator should be greater than 0.140, and a sample was negative if the OD sample/OD calibrator ratio was less than 0.80, a sample was positive if the OD sample/OD calibrator ratio was greater than 1.10, and a sample was indeterminate if the OD sample/OD calibrator ratio was between 0.80 and 1.10. In the latter case, the test should be repeated. For all negative samples, a rubella serology test should be performed afterward.

### 2.6. Statistical Analysis

All measles cases in the analysis were laboratory-confirmed. The epidemiology of cases was performed using the following variables: age, sex, vaccination status, area of residence, and department. Age was categorized into classes (0–5 years old, 5–9 years old, and 10 years and above). Sex was grouped into male and female. Vaccination status was categorized as unvaccinated, vaccinated with one dose, vaccinated with two doses, did not know, and not reported. Housing areas were classified as urban and rural. In addition, the study covered the entire country, and the department variable was taken into account to cover the reporting areas where the focal points were based.

### 2.7. Analytical Approach

Patient data and laboratory results were recorded using Epi Info Version 3.5.4 or Microsoft Excel 2010. We used also Microsoft Excel 2010 for the extraction of the data and description of the study population based on the calculation of proportions and percentages. R software Version 4.2 and GraphPad Version 8.0 were used to calculate risk factors and determine the effect of each variable on the probability of contracting measles (e.g., the effect of age on the risk of getting measles). For this purpose, adjusted odds ratios (aORs) were determined by performing a univariate logistic regression calculation with the adjustment of each variable: independent (age, sex, vaccination status, etc.) and dependent (confirmed cases). To estimate the precision of each aOR, 95% confidence intervals were issued. The *p* value of each aOR was also calculated to determine whether each independent variable was significantly associated with the independent variable (these differences were statistically significant when the *p* value was below the 0.05 threshold).

### 2.8. Ethical Approval and Consent to Participate

The samples were obtained through the measles surveillance system. As this infection is notifiable, an ethical agreement was not needed. However, a statement summarizing the objectives of the surveillance was read to each parent in French or in one of the two national languages (Lingala and Kituba). The interviews were conducted in private to guarantee the confidentiality of the information collected, in accordance with the Declaration of Helsinki (World Medical Association [WMA]).

## 3. Results

### 3.1. Sociodemographic Characteristics of all Laboratory-Confirmed Measles Cases (RoC, 2019–2022)

A total of 1330 cases were recorded from January 2019 to October 2022, where the mean age was 05 years (3–11 years). Children under 05 years of age (49.5%) were the most represented in this study population. We recorded 673 (50.6%) male patients and 647 (48.6%) female patients. Most of the notified cases lived in urban areas: 919 (69.1%). The cities of Pointe-Noire and Brazzaville reported the most cases, with 292 (22%) and 256 (19.2%), respectively. The vaccination rate was very low. Indeed, among the patients whose vaccination status was known, only 430 (32.3%) had received a single dose of the vaccine compared to 166 (12.5%) who had received two doses. This resulted in an overall vaccination rate of 44.8%. A total of 526 children (39.6%) were not vaccinated. It was also noted that 5.3% of patients did not know their vaccination status, while 137 (10.3%) patients did not report their vaccination status (Table 1).

### 3.2. Description of Laboratory-Confirmed Positive Measles Cases (RoC, 2019–2022)

Of the 1330 samples tested, 537 samples (40.3%) were laboratory-confirmed to be positive. The highest rate of positivity was observed in 2022, with 65.9% compared to 16.8% in 2019 (Table 2). A high rate was observed not only in unvaccinated (46.6%) but also in both one-dose and two-dose vaccinated children (36.75%). The age group analysis revealed that the proportion of children under 5 years old was the highest (44.6%), followed by the 5–9-years-old group (40.8%). During this period, the highest rate was observed in Likouala (68.6%) and Pool (66.6%), while the lowest was reported in the Cuvette department (20.5%) followed by Brazzaville (26.5%) (Table 2).

**Figure 2:**
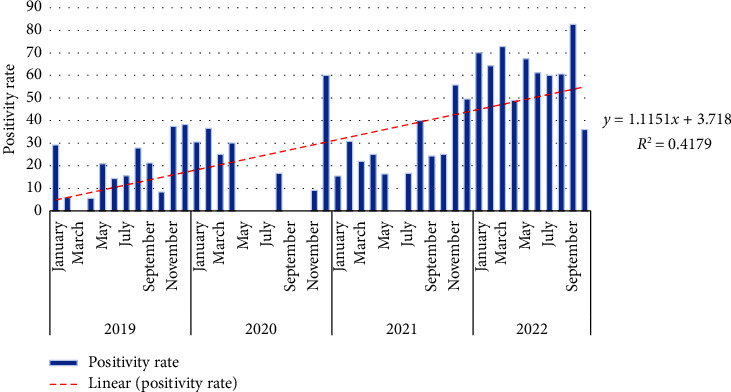
Trend of laboratory-confirmed positive measles laboratory cases by month (RoC, 2019–2022).

Figure 2 shows the cumulative evolution of positive cases per year. A progressive evolution of positive cases was noted, although, with a notable increase of cases in the last quarter of each year. An outbreak and progressive evolution of positive confirmed cases was observed during the study, going from 16.8% in 2019 to 65.9% in 2022.

### 3.3. Incidence Per 100,000 Laboratory Measles Cases in Different Departments (RoC, 2019–2022)

**Figure 3:**
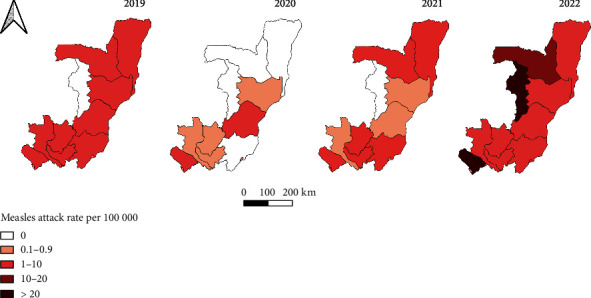
Geographical distribution of measles in the Republic of the Congo from 2019 to 2022. The attack rate per 1,00,000 population in each map is categorized into 0, 0.1–0.9, 1–10, 10–20, and > 20. Maps were designed using QGIS software. The source file was downloaded to GADM online.

The departments of Brazzaville, Pointe-Noire, and Bouenza, which were the most populous departments, had a high proportion of positive cases but had the lowest incidence during this period. High incidence was found in Cuvette-Ouest, Likouala, and Kouilou departments bordered by endemic countries (Figure 3 and Table 3).

### 3.4. Risk Factors

In Table 4, we report the exposure factors for measles. Females were slightly more exposed to the disease than males (*p*=0.04; aOR: 1.25 [1.01–1.6]) and the risk of getting measles was high among people living in rural areas (*p* < 0.0001). Three departments were considered as high-risk areas. These were Likouala (*p*=0.0001; aOR: 3.18 [1.80–5.61]), Pool (*p*=0.0001; aOR: 2.90 [1.70–4.95]), and Brazzaville (*p*=0.0005; aOR: 0.52 [0.36–0.75]). Overall, lack of vaccination was identified as a risk of exposure to infection (*p*=0.0008). However, no difference was observed between unvaccinated individuals and those who received two doses (*p*=0.943).

## 4. Discussion

The purpose of this study was to describe the epidemiological characteristics of notified measles cases in the Republic of the Congo from January 2019 to October 2022 as well as the risk factors associated with the occurrence of measles in a particular context of post and between COVID-19 outbreak. We report an overall proportion of 40.3% of positive laboratory cases for these 4 years. The highest proportion was observed in 2022, resulting from a steady increase in the number of cases since 2019. The departments of Brazzaville, Likouala, Pool, and Pointe-Noire were the most affected based on the positivity rate. This circulation does not appear to be seasonal, as no differential trend was observed at any time of the year. This observation seems to corroborate a study conducted in Bauchi State in Nigeria, in which continuous circulation of the virus was observed throughout the year [18]. However, this circulation in our study shows a significant slowdown in 2020, probably as a consequence of the prevention and control measures imposed during the COVID-19 pandemic. These restrictive measures severely disabled certain activities related to epidemiological surveillance and overall attendance at health facilities during this pandemic. It has also been emphasized that resources have decreased, as they have been prioritized for the response to COVID-19 in recent years [19]. This observation is consistent with the findings of several studies that have noted a direct and/or indirect impact of the COVID-19 pandemic in reducing measles eradication efforts [20–22]. In our case, this factor would probably have not only muted the progressive increase in measles cases but also favored its spread due to the absence of corrective measures. Indeed, it is demonstrated in our study that as soon as these restrictive measures were removed, notably the lockdown of populations and the limitation of interdepartmental travel, a significant increase in measles cases was observed as early as 2021, thus contrasting with what had been observed in our country for nearly a decade.

In the present study, a particularly low vaccination rate was observed (44.81%), and only 12.5% of people had a complete vaccination schedule. These vaccination rates, which are far below the eradication rate recommended by the WHO (95%), probably explain the high number of infections in our study population (40.3%). In addition, the data reported here raise questions about the effectiveness of the vaccination strategies in our context, due to the large number of patients affected among those who received one or two doses of the vaccine. Moreover, there was no discernible difference between patients who received one or two doses of the vaccine. Although similar data on vaccine coverage have been reported in Gambia and Nigeria [14, 23], the data we report could also be explained by a lack of cold chain compliance and management during vaccination operations. The type of vaccine administered could also explain these findings. It has been shown that one dose of vaccine induces a better immune response and a significantly higher antibody titer with monovalent vaccines than with combined vaccines [24]. In the Republic of the Congo, a combined vaccine is administered as a part of the vaccination strategy adopted since 2015. However, this conclusion could be relativized as there is evidence that host-specific immune factors could be at the origin of these observations in vaccinated individuals. Indeed, some studies report that 10% of people who received two doses of combined MMR vaccine would still develop measles [25, 26]. Indeed, in a comparative study, polymorphisms in interleukins (IL-4/IL-13) in Mozambican children were shown to affect the immune response to measles vaccines compared to Australian children in whom this genetic variability was not observed [25]. Similar results were observed in China, where high morbidity was observed in different study populations reporting a vaccination history [26, 27]. In our study, we reported a low correlation rate related to sex (*p* < 0.04). Indeed, females were at greater risk of measles than males despite a sex ratio tilted in favor of males. These results differ from those usually observed in the literature [14, 28]. A proportion of over 40% has been reported regardless of the age group considered. As indicated above, this observation is the result of incomplete immunization of the target population or of a fringe of the population that, despite having received two doses, is still exposed to the disease, even though these percentages are much higher than 10%, as is usually accepted. These observations have also been made in other studies reporting the vaccination gap in similar study populations [29–31]. Spatially, the departments of Brazzaville, Pointe-Noire, and Bouenza had high proportions of positive cases but low incidence. This could be explained by the fact that these departments are the most populated in the country. Departments bordering endemic countries such as Likouala and Kouilou reported high incidences. The mixing of people, where there are numerous exchanges with populations of different origins, may be conducive to the spread of this infection. Similar results have been observed in South Africa [13].

## 5. Conclusion

Measles remains a major public health problem in the Republic of the Congo. In view of the results, it is becoming urgent to strengthen the vaccination strategy not only among children under 5 years of age but also among those of school age to reduce the vaccination gap observed in the various age groups. Particular attention should not only be given to rural areas but also to the departments of Pointe-Noire, Brazzaville, and Bouenza. Indeed, studies should be conducted to better understand factors that lead to a high positive rate among vaccinated persons. Also, we recommend to the Ministry of Health, through the Expanded Program of Immunization, to re-evaluate the use of a monovalent measles vaccine or a combined vaccine for the elimination of this disease in the Republic of the Congo.

## Figures and Tables

**Table 1:** General characteristics of suspected measles cases referred to the laboratory in RoC from 2019 to 2022.

Variables	*N* = 1330	2019 *n* (%)	2020 *n* (%)	2021 *n* (%)	2022 *n* (%)
Age groups *n* (%)					
< 5	659 (49.5)	88 (30.2)	84 (59.6)	224 (52.7)	263 (55.6)
5–9	372 (28)	94 (32.3)	39 (27.7)	126 (29.6)	113 (23.9)
≥10	195 (14.7)	38 (13.1)	15 (10.6)	53 (12.5)	89 (18.8)
Missed	104 (7.8)	71 (24.4)	3 (2.1)	22 (5.2)	8 (1.7)
Sex *n* (%)					
Males	673 (50.6)	151 (51.9)	75 (53.2)	211 (49.6)	238 (50.3)
Females	647 (48.6)	135 (46.4)	66 (46.8)	211 (49.6)	233 (49.3)
Missed	10 (0.8)	5 (1.7)	0 (0)	3 (0.8)	2 (0.4)
Vaccination status					
Unvaccinated	526 (39.6)	100 (34.4)	65 (46.1)	130 (30.6)	231 (48.8)
One dose	430 (32.3)	125 (42.9)	53 (37.6)	127 (29.9)	125 (26.4)
Two doses	166 (12.5)	29 (10)	10 (7.1)	89 (20.9)	38 (8)
Unknown	71 (5.3)	19 (6.5)	3 (2.1)	18 (4.2)	31(6.6)
Missed	137(10.3)	18 (6.2)	10 (7.1)	61 (14.4)	48 (10.2)
Regions					
Brazzaville	256 (19.2)	57 (19.6)	25 (17.7)	76 (17.9)	98 (20.7)
Pointe-Noire	292 (22)	84 (28.9)	45 (31.9)	129 (30.4)	34 (7.2)
Sangha	44 (3.3)	12 (4.1)	2 (1.4)	13 (3.05)	17 (3.6)
Likouala	67 (5.1)	16 (5.5)	4 (2.8)	21 (4.9)	26 (5.4)
Niari	71 (5.3)	7 (2.4)	6 (4.3)	15 (3.5)	43 (9.1)
Kouilou	95 (7.1)	13 (4.5)	11 (7.8)	24 (5.6)	47 (10)
Bouenza	143 (10.8)	25 (8.6)	17 (12.1)	43 (10.1)	58 (12.3)
Plateaux	68 (5.1)	17 (5.8)	9 (6.4)	27 (6.4)	15 (3.2)
Lékoumou	64 (4.8)	12 (4.1)	13 (9.2)	22 (5.2)	17 (3.6)
Pool	75 (5.6)	16(5.5)	0 (0)	13 (3.05)	46 (9.7)
Cuvette	73 (5.5)	22 (7.6)	7 (5.0)	28 (6.6)	16 (3.4)
Cuvette-Ouest	82 (6.2)	10 (3.4)	2 (1.4)	14 (3.3)	56 (11.8)
Municipality					
Urban area	919 (69.1)	222 (76.3)	117 (83.0)	287 (67.5)	293 (61.9)
Rural area	411 (30.9)	69 (23.7)	24 (17.0)	138 (32.5)	180 (38.1)

Abbreviation: %, percentage; *n*, number per year; *N*, total number.

**Table 2:** Positivity rate of confirmed positive measles cases by department (RoC, 2019–2022).

Departments	All participants		2019		2020		2021		2022	
	Suspected cases	Positive IgM (%)	Suspected cases	Positive IgM (%)	Suspected cases	Positive IgM (%)	Suspected cases	Positive IgM (%)	Suspected cases	Positive IgM (%)
Pointe-Noire	292	119 (40.7)	84	3 (3.6)	45	11 (24.4)	129	94 (72.8)	34	11 (32.3)
Brazzaville	256	68 (26.5)	57	6 (10.5)	25	6 (24)	76	3 (3.9)	98	53 (54.0)
Kouilou	95	44 (46.3)	13	2 (15.3)	11	3 (27.2)	24	2 (8.3)	47	37 (78.7)
Bouenza	143	60 (41.9)	25	5 (20)	17	3 (17.6)	43	5 (11.6)	58	47 (81.0)
Niari	71	33 (46.4)	7	1 (14.2)	6	2 (33.3)	15	1 (6.6)	43	29 (67.4)
Lekoumou	64	22 (34.3)	12	2 (16.6)	13	1 (7.6)	22	5 (22.7)	17	14 (82.3)
Pool	75	50 (66.6)	16	9 (56.2)	0	0 (0)	13	5 (38.4)	46	36 (78.2)
Plateaux	68	28 (41.1)	17	8 (47.0)	9	8 (88.8)	27	2 (7.4)	15	10 (66.6)
Cuvette	73	15 (20.5)	22	4 (18.1)	7	2 (28.5)	28	2 (7.1)	16	7 (43.7)
Cuvette-Ouest	82	32 (39.0)	10	0 (0)	2	0 (0)	14	0 (0)	56	32 (57.1)
Sangha	44	20 (45.4)	12	2 (16.6)	2	0 (0)	13	4 (30.7)	17	14 (82.3)
Likouala	67	46 (68.6)	16	7 (43.7)	4	0 (0)	21	17 (80.9)	26	22 (84.6)

**Table 3:** Incidence per 100.000 of laboratory measles cases in different departments (RoC, 2019–2022).

Departments	Total positives of laboratory measles cases	Proportion (%)	Population∗	Incidence per 100.000
Pointe-Noire	119	22.2	1,302,577	9.1
Brazzaville	68	12.7	2,113,112	3.2
Kouilou	44	8.2	1,17,440	37.5
Bouenza	60	11.2	4,81,595	12.5
Niari	33	6.1	3,60,354	9.2
Lekoumou	22	4.1	1,50,225	14.6
Pool	50	9.3	3,98,103	12.6
Plateaux	28	5.2	2,72,048	10.3
Cuvette	15	2.8	2,43,256	6.2
Cuvette-Ouest	32	5.9	1,13,749	28.13
Sangha	20	3.7	1,33,726	15
Likouala	46	8.6	2,40,168	19.15
Total	537		5,926,353	

^∗^Source: Republic of the Congo. Ministry of Health and Population/Directorate of Epidemiology and Disease Control/Expanded Program on Immunization: National Plan of Response to the Measles Epidemic in Congo. March 2022 (17).

Abbreviation: *N*, confirmed cases.

**Table 4:** Risk factors associated with confirmed measles cases (RoC, 2019–2022).

Variables	Suspected cases, *n*	Confirmed cases, *n* (%)	aOR adjusted (95% CI)	*p* value
Age groups				
< 5	659	294 (44.6)	Reference	
5–9	372	152 (40.8)	0.85 (0.66–1.10)	0.2429
≥10	195	79 (40.5)	0.84 (0.61–1.16)	0.3108
Sex				
Males	673	253 (37.6)	Reference	
Females	647	279 (43.1)	1.25 (1.01–1.56)	0.0407
Vaccination status				
Unvaccinated	526	245 (46.6)	Reference	
One dose	430	154 (35.8)	0.64 (0.49–0.83)	0.0008
Two doses	166	65 (39.2)	0.73 (0.51–1.05)	0.0943
Departments				
Pointe-Noire	292	119 (40.7)	Reference	
Brazzaville	256	68 (26.5)	0.52 (0.36–0.75)	0.0005
Kouilou	95	44 (46.3)	1.25 (0.78–1.99)	0.3407
Bouenza	143	60 (41.9)	1.05 (0.70–1.57)	0.8105
Niari	71	33 (46.4)	1.26 (0.74–2.12)	0.3810
Lékoumou	64	22 (34.3)	0.76 (0.43–1.34)	0.3456
Pool	75	50 (66.6)	2.90 (1.70–4.95)	0.0001
Plateaux	68	28 (41.1)	1.01 (0.59–1.74)	0.9490
Cuvette	73	15 (20.5)	0.37 (0.20–0.69)	0.0018
Cuvette-Ouest	82	32 (39.0)	0.93 (0.56–1.53)	0.7780
Sangha	44	20 (45.4)	1.21 (0.64–2.29)	0.5554
Likouala	67	46 (68.6)	3.18 (1.80–5.61)	0.0001
Municipality				
Urban	919	252 (27.4)	Reference	
Rural	411	195 (47.4)	0.41 (0.32–0.53)	< 0.0001

## Data Availability

The data used to support the findings of this study are available from the corresponding author upon request.

## Nomenclature

aOR: Adjusted odds ratio
COVID-19: Coronavirus disease 2019
DRC: Democratic Republic of Congo
OD: Optical density
EDTA: Ethylenediaminetetraacetic acid
ELISA: Enzyme-linked immunosorbent assay
CI: Confidence interval
IL-4/13: Interleukins 4/13
*p* or *p* value: Probability value
RoC: Republic of the Congo
WHO: World Health Organization.
